# Factors Associated with Quality of Life Among Patients with Cardiac Pacemakers Assessed by Two Scales

**DOI:** 10.3390/clinpract16030053

**Published:** 2026-02-28

**Authors:** Eirini Stavrou, Georgios Vasilopoulos, Dionyssios Leftheriotis, Panagiota Flevari, Maria Polikandrioti

**Affiliations:** Applied Clinical Nursing Postgraduate Program, Nursing Department, University of West Attica, 12243 Egaleo, Greece

**Keywords:** cardiac device, quality of life, pacemaker

## Abstract

**Background/Objectives**: Permanent cardiac pacemakers (PPMs) are small electronic implanted devices that regulate cardiac rhythm. Measurement of quality of life (QoL) serves as a powerful tool for gaining in-depth insights into pacing therapy and ultimately guiding patient-centered management strategies. The aim of the present study was to evaluate factors affecting QoL among PPM patients by applying the two generic questionnaires: SF-36 and EQ-5D-5L. **Materials and Methods**: A total of 120 patients with PPM were enrolled. QoL data were collected through interviews using the 36-Item Short Form Health Survey (SF-36) and the Euro QoL 5-Dimensions 5-Levels Health Questionnaire (EQ-5D-5L). Patients’ characteristics were also recorded. **Results**: The majority of participants were male (54.2%), retired (83.3%) residents in urban areas (75.5%), had a DDD pacemaker (82.5%), had rate response programmed on (77.5%), and had comorbidities (83.3%). Regarding QoL measured by SF-36, the Physical Component Summary Score (PCS) was significantly associated with programming rate response in their pacemaker (*p* = 0.046), comorbidities (*p* = 0.047), and the NYHA functional class (*p* = 0.047). The Mental Component Summary Score (MCS) was significantly associated with sex (*p* = 0.034), place of residence (*p* = 0.003), NYHA functional class (*p* = 0.001), and patients’ level of information about the device (*p* = 0.039). Patients’ QoL, as measured by the EQ-5D-5L, was significantly associated with sex (*p* = 0.001), age (*p* = 0.019), occupation (*p* = 0.040), pacing mode (*p* = 0.034), comorbidities (*p* = 0.019), NYHA functional class (*p* = 0.047), and level of information about the device (*p* = 0.005). **Conclusions:** NYHA functional class, comorbidities, and level of information as reported by patients were the factors associated with QoL, as shown by the two scales. All three factors guide a personalized care plan since NYHA class shows the burden of disease, comorbidities add to the complexity, and patient information determines the effectiveness of management.

## 1. Introduction

During recent decades, the implantation of permanent cardiac pacemakers (PPMs) has been steadily increasing, driven by advances in device technology, improvements in cardiovascular practice, better diagnostic methods, and broader clinical indications [1,2]. It is estimated that 1.25 million PPMs are implanted every year worldwide [3]. In the United States, approximately 250,000 new PPMs are placed annually [4]. In Greece, the annual number of implants is 895.8 devices per million inhabitants (approximately 8958 devices), according to recent data from the European Society of Cardiology (2020) [5].

PPM implantation represents an essential cardiological intervention in the management of bradyarrhythmias and conduction abnormalities. Evidence-based guidelines have expanded the indications for PPM implantation, including certain cases of heart failure, atrioventricular block, and sinus node dysfunction [6]. PPM implantation rates increase with age, as 70–80% of recipients are over 65 years of age [7]. For example, in Norway, at the time of implantation, the majority of recipients were >60 years old and approximately 40% were ≥81 years old [8]. In the foreseeable future, the PPM implantation rate is anticipated to increase due to the demographic shift towards an aging population. Astonishingly, global projections indicate that the population aged over 60 will rise from 1 billion in 2020 to 1.4 billion by 2030, while the number of individuals aged 80 and above is expected to triple between 2020 and 2050, reaching approximately 426 million [9].

Τhe ultimate goal of PPM implantation is to reduce symptoms of disease (dizziness, fainting, fatigue), prevent arrhythmia-related complications (heart failure, stroke, sudden cardiac death), and improve recipients’ quality of life (QoL) [10]. Interestingly, the technological advancements alone may not ensure optimal patient outcomes; therefore, QoL measurement enhances clinicians’ awareness of patient priorities, physical and mental health, or daily functioning. The World Health Organization stated that QoL is the “individual’s perception of his or her position in life in the context of the culture and value system in which he or she lives and in relation to his or her goals, expectations, standards, and concerns” [7]. Accordingly, QoL assessment is a key element for providing patient-centered care since it illustrates experiences in the recipient’s daily life, and may unmask gaps between reality and their hopes and expectations [11]. Several instruments have been developed to measure QoL, including the 36-Item Short Form Health Survey (SF-36) and the Euro QoL 5-Dimensions 5-Levels Health Questionnaire (EQ-5D-5L) [7].

Current knowledge regarding PPM recipients illustrates various factors associated with QoL, such as demographics (age, sex, and socio-economic status), clinical status (comorbidity, as well as early and late complications), and patients’ perceptions about device efficacy and benefits [2,11,12]. Obviously, device implantation is a major life change that may affect the physical, mental, and functional state of patients. Therefore, the rationale of this study was to explore QoL by the combined use of two scales, which may allow researchers and clinicians to obtain a more descriptive profile of health status among PPM recipients. Utilizing both research instruments detects impairments across dimensions, captures overall health status, and facilitates comparative QoL analysis, thus enforcing evaluation of patient-centered outcomes [13].

The objective of this study was to evaluate factors associated with QoL among PPM patients by applying the two generic questionnaires: SF-36 and EQ-5D-5L.

## 2. Materials and Methods

### 2.1. Design, Setting, and Period of the Study

In this cross-sectional and single-center study, 120 patients with implanted PPMs who attended scheduled follow-up visits at the outpatient clinic of a public hospital in Attica, from 10 March 2025 to June 2025, were enrolled. Initially, 140 patients were approached, but 20 refused. Participants were selected by the method of convenience sampling.

According to this selection method, participants were enrolled based on their proximity and availability and not through a random selection process (non-probability sampling method). This is a well-known approach in healthcare research and cardiovascular nursing, despite the risk of bias and generalizability of results [14].

*Description of the sample:* PPM recipients had either a pacemaker of Ventricular Pacing, Ventricular Sensing, Inhibited Response (VVI), or a Dual-chamber Pacing, Dual-Chamber Sensing, Dual Response (DDD). VVI uses a single lead placed in the right ventricle and delivers an impulse when it does not sense a natural ventricular beat. DDD has two leads: one is placed in the right atrium and one in the right ventricle and paces the atrium, the ventricle, or both as needed, depending on intrinsic activity and maintaining atrioventricular synchrony [15].

### 2.2. Inclusion and Exclusion Criteria of the Sample

Criteria for inclusion in the study were: (a) understanding the Greek language and (b) the ability to comprehend, read, and sign the informed consent form. Exclusion criteria were: (a) insufficient communication abilities, (b) sensory impairment (hearing or seeing), and (c) having a leadless PPM. Other exclusion criteria provided by the medical record or by the cardiologist were diagnoses of (a) psychiatric comorbidity and receiving medical treatment, and/or (b) cognitive impairment, as these may confound the results.

### 2.3. Data Collection and Procedure

Data collection was performed for each participant after device interrogation and clinical follow-up and lasted approximately 20 min for each patient. The procedure was carried out by the method of interview to avoid missing data and in a private office at the outpatient clinics to ensure confidentiality and privacy.

### 2.4. Research Instrument

Data collection was carried out using the following standardized scales to measure QoL: a. The SF-36 Health Survey (SF-36) and b. The Euro QoL 5-Dimensions 5-Levels Health Questionnaire (EQ-5D-5L). Data collected for each patient also included demographic and clinical characteristics, as well as other self-reported variables, which were selected through clinical observations and literature review.

#### 2.4.1. Assessment of QoL by SF-36 Health Survey (SF-36)

The SF-36 Health Survey (SF-36) was used to evaluate QoL. The SF-36, developed by Ware and colleagues in 1993, assesses both physical and mental health. It consists of 36 items grouped into eight dimensions: Physical Functioning, Role-Physical, Role-Emotional, Bodily Pain, General Health, Vitality, Social Functioning, and Mental Health. Respondents answer using Likert-type scales. Scores for each dimension are calculated separately. Additionally, two summary scores are derived: the Physical Component Summary (PCS) and the Mental Component Summary (MCS), which can be compared with population norms. The total scores range from 0 to 100, with higher scores indicating better QoL [16,17]. Cronbach’s alpha values reported for the SF-36 Greek version subscales ranged approximately from 0.70 to 0.90, indicating acceptable to excellent internal consistency and confirming the hypothesized scale structure via multitrait scaling. Intraclass Correlation Coefficient (ICC) values for test–retest reliability were reported generally above 0.70, supporting good reproducibility. MCID is the smallest meaningful change in SF-36 scores, usually 3–5 points for summary scores. It helps determine if health changes are clinically important [17].

#### 2.4.2. Assessment of QoL by EQ-5D-5L

The EQ-5D-5L is a standardized instrument developed by the EuroQol Group to measure health-related QoL. It consists of two parts: a. a descriptive system of health status across five dimensions—mobility, self-care, usual activities, pain/discomfort, and anxiety/depression—each with five levels of severity, and b. visual analog scale (EQ VAS) ranging from 0 to 100, where individuals rate their overall current health status. Each combination of responses generates a five-digit code (e.g., 12345), which corresponds to a unique utility value based on country-specific value sets derived from population studies. Utility values range theoretically from −0.66 (worse than death) to 1.00 (perfect health), depending on the specific country’s value set. The combination of the descriptive system and EQ VAS provides a comprehensive picture of the individual’s perceived quality of life [18,19]. There is good test–retest reliability for the EQ-5D-5L dimensions. Agreement statistics (like weighted kappa) ranged generally from 0.60 to 0.90, indicating moderate to excellent stability of responses over time [18]. The EQ-5D-5L has five distinct dimensions (mobility, self-care, usual activities, pain/discomfort, anxiety/depression), each measured by a single item. The scale has been validated in Greek, demonstrating good psychometric properties, i.e., high internal consistency; Cronbach’s alpha coefficient was 0.884 [19].

### 2.5. Ethical Considerations

The present study was approved by the Research Committee of the public hospital (Number: 117/4 February 2025). Participants were informed by the researcher of the purpose of this study, and their written informed consent was obtained. Data collection guaranteed anonymity and confidentiality. All subjects were informed of their rights to refuse or discontinue participation in the study, according to ethical standards of the Declaration of Helsinki (1989) of the World Medical Association.

### 2.6. Statistical Analysis

Categorical data are presented as absolute and relative frequencies (%), while continuous data are presented as mean, standard deviation (SD), median, and interquartile range (IQR). The *t*-test and ANOVA, or Kruskal–Wallis and Mann–Whitney tests, were used to examine the association between QoL scores and patient characteristics, depending on whether the data followed a normal distribution or not, respectively. Normality was assessed using the Shapiro–Wilk test and visually through histograms. Multiple linear regression analysis was used to estimate the effect of patient characteristics on QoL. Results are presented with β regression coefficients and 95% confidence intervals (CIs). A *p*-value of less than 0.05 was considered statistically significant. All statistical analyses were performed using SPSS software, version 25 (SPSS Inc., Chicago, IL, USA).

## 3. Results

### 3.1. Sample Description

According to Table 1, the majority of participants were male (54.2%). Slightly more than half (51.7%) were 75–84 years old, 83.3% of patients were retired, and 75.5% resided in Athens or another urban area. According to Table 2, the majority had a DDD pacemaker (82.5%), had rate response programmed on (77.5%), had comorbidities (83.3%), were NYHA class I (74.2%), had a family member with chronic illness (31.7%), believed their life depended on the device (82.5%), declared themselves “very well and fairly” informed about their device (88.4%) and had a check-up once a year (87.5%).

### 3.2. QoL Results

Table 3 presents the results regarding patients’ QoL as measured by the SF-36 scale. More in detail, in the SF-36 scale, the summary scores of physical and mental components suggest that patients had below-average physical health compared to the general population (median PCS score: 39, below the reference value of 50), and above-average mental health (median MCS score: 58.9, above 50). Overall, the patients’ physical health was worse than their mental health. In terms of the eight dimensions in ascending order, from the minimum mean ± SD to the maximum, results showed: Physical Functioning (23.3 ± 16.4), Role-Physical (57.3 ± 21.6), Bodily Pain (64.8 ± 24.9), Mental Health (73.9 ± 23.9), General Health (74.2 ± 43.6), Role-Emotional (75.0 ± 42.4), Social Functioning, (75.0 ± 28.9) and Vitality (85.3 ± 20.8). Cronbach’s a values indicate high internal consistency of participants’ answers (Cronbach’s a > 0.7).

Table 4 and Table 5 describe QoL with the EQ-5D-5L scale. Patients’ QoL was high, with a median utility score of 0.9 (where the maximum possible value is 1). Regarding the five dimensions of the scale, the proportions of patients reporting more than moderate problems were as follows: Mobility: 31.7%, Anxiety/Depression: 29.2%, Usual Activities: 28.3%, Pain/Discomfort: 21.7%, and Self-Care: 7.5%. Cronbach’s a value indicates high internal consistency of participants’ answers (Cronbach’s a = 0.848).

### 3.3. Factors Associated with QoL by SF-36

Table 6 presents the associations between patient characteristics and their QoL as assessed by the SF-36 scale.

The Physical Component Summary Score (PCS) was significantly associated with the rate response in their pacemaker (*p* = 0.046), comorbidities (*p* = 0.047), and the NYHA functional class (*p* = 0.047). Specifically, those without rate response had significantly lower PCS (median = 35.2) compared to those with rate response (median = 39.0). Patients with comorbid conditions had worse PCS (median = 35.7) than those without (median = 39.2). Patients classified as NYHA class II had significantly lower PCS (median = 34.5) compared to those in NYHA class I (median = 39.4).

The Mental Component Summary Score (MCS) was significantly associated with sex (*p* = 0.034), place of residence (*p* = 0.003), NYHA functional class (*p* = 0.001), and patients’ level of information about the device (*p* = 0.039). Specifically, female patients had worse MCS (median = 56.3) compared to male patients (median = 62.1). Patients living in Athens or urban areas had lower MCS (median = 56.4) compared to those living in rural areas (median = 64.8). Patients in NYHA class II reported significantly lower MCS (median = 46.8) compared to those in class I (median = 62.2). Patients who self-reported being fairly or very well informed about the device had higher MCS (medians = 60.2 and 58.9, respectively) than those who felt poorly informed (median = 49.7).

### 3.4. Factors Associated with QoL by EQ-5D-5L

Table 7 presents the associations between patients’ characteristics and their QoL as assessed by the EQ-5D-5L scale. Patients’ QoL, as measured by the EQ-5D-5L, was significantly associated with sex (*p* = 0.001), age (*p* = 0.019), occupation (*p* = 0.040), type of pacemaker (*p* = 0.034), comorbidities (*p* = 0.019), NYHA functional class (*p* = 0.047), and level of information about the device (*p* = 0.005). More specifically, male patients reported significantly better QoL (median = 0.9) compared to female patients (median = 0.8). Patients under the age of 85 had better QoL (median = 0.9) compared to those over 85 (median = 0.85). Employed patients had the highest QoL (median = 1.0) compared to retired (median = 0.9) and unemployed patients (median = 0.8). Patients with a VVI pacemaker reported significantly worse QoL (median = 0.7) than those with a DDD pacemaker (median = 0.9). Those with comorbid conditions had worse QoL (median = 0.8) than those without comorbidities (median = 1.0). Patients in NYHA class II had worse QoL (median = 0.6) compared to those in class I (median = 0.9). Additionally, patients who felt poorly informed about the device had significantly lower QoL (median = 0.7) than those who felt very or fairly well informed (median = 0.9).

All association are presented in Table 8.

### 3.5. Effect of Patient Characteristics on QoL

In Table 9, a multiple linear regression analysis was conducted to estimate the effect of patient characteristics (independent variables) on their QoL (dependent variable).

Regarding the Physical Component of QoL (SF-36), patients classified as NYHA class II had significantly lower scores (by 3.3 points) compared to those in NYHA class I (β = −3.33, 95% CI: −6.43 to −0.23, *p* = 0.036). For the Mental Component of QoL (SF-36), female patients had significantly lower scores (by 5.8 points) compared to male patients (β = −5.84, 95% CI: −10.54 to −1.15, *p* = 0.015). Patients living in rural areas had significantly higher mental health scores (by 10 points) compared to those living in Athens/urban area (β = 10.06, 95% CI: 4.40 to 15.73, *p* = 0.001). Additionally, patients in NYHA class II had lower mental health scores (by 10.9 points) than those in class I (β = −10.88, 95% CI: −16.48 to −5.27, *p* = 0.001).

As for overall QoL measured by the EQ-5D-5L, patients with a DDD pacemaker had significantly higher utility scores (by 0.12) compared to those with a VVI pacemaker (β = 0.12, 95% CI: 0.01 to 0.23, *p* = 0.027). Patients in NYHA class II had lower EQ-5D-5L scores (by 0.20) compared to those in class I (β = −0.20, 95% CI: −0.29 to −0.11, *p* = 0.001). Lastly, patients who felt poorly informed about their device had 0.23 points lower quality of life scores than those who felt very well informed (β = −0.23, 95% CI: −0.36 to −0.11, *p* = 0.001).

## 4. Discussion

In relation to the objective of identifying factors associated with QoL, the Mental Component Summary (MCS) of the SF-36 was significantly associated with sex, place of residence, NYHA functional class, and patients’ level of information about the device while the Physical Component Summary Score (PCS) was significantly associated with programming rate response in their pacemaker, comorbidities and the NYHA functional class. With respect to the objective of determining factors associated with overall QoL measured by the EQ-5D-5L, a significant association was found with sex, age, occupation, pacing mode, comorbidities, NYHA functional class, and patients’ level of information about the device. These findings suggest that QoL is shaped by a combination of demographic, clinical, and device-related factors.

While both are widely used to assess QoL across medical conditions, they differ in structure, sensitivity, and focus. Specifically, the EQ-5D-5L measures five QoL dimensions: mobility, self-care, usual activities, pain, discomfort, and anxiety/depression. The SF-36 includes eight domains covering both physical and mental health aspects in detail, making it particularly sensitive to differences in mental health and psychosocial functioning. The instruments differ in item number, assessed health dimensions, output format, and intended use. When two instruments evaluate the same outcome (QoL) and produce consistent results, then this concordance enhances the credibility of the research findings and reinforces their utility for implementation of policy strategies and clinical decision-making [13].

As measured by two scales, worse QoL was observed in participants with comorbid conditions and NYHA class II. A higher NYHA class reflects more severe symptoms, while comorbidity adds to the disease burden, increasing functional limitations, hospitalizations, and emotional distress. It is widely known that the NYHA classification is based on symptoms, mainly dyspnea, fatigue, palpitations, or angina, in relation to physical activity [20]. Fatigue significantly impairs patients’ capacity to manage daily activities, self-care, and treatment adherence, often leading to decreased social engagement and subsequent isolation [21].

Patients with comorbidities frequently encounter challenges largely attributable to the complexity of treatment regimens and polypharmacy. Likewise, these individuals are more likely to engage in vigilant health monitoring to avoid any risk of clinical instability^.^ Higher comorbidity burden correlates with lower baseline QoL and predicts a subsequent relative decline. Comorbidity is associated with increased mortality and hospitalization rates; however, its role in predicting changes in QoL over time remains underexplored. Notably, individuals with comorbidities are underrepresented in clinical trials [22,23]. As a consequence, trial results may not fully reflect real-world patient populations, reducing the applicability of evidence to clinical practice and creating uncertainty about effective treatments to improve QoL.

Participants who felt poorly informed about the device had worse MCS and QoL as measured by the EQ-5D-5L scale. Indeed, inadequate access to information leads to limited comprehension of the therapy, ambiguities, and misunderstandings, which are an obstacle in adapting to life with a PPM [2]. In Greece, a relevant study among 150 PPM recipients demonstrated that 18% reported being “little or no” informed about therapy, and 15.3% believed that the device would prevent disease progression [2]. In the same study, well-informed PPM recipients had a better QoL in physical and emotional roles, social functioning, and general health. Interestingly, well-informed patients tend to collaborate more effectively with clinicians, demonstrate greater self-care, and are less likely to adopt unreliable treatment practices. These behaviors, in turn, positively influence their QoL [2]. In the same context, a noticeable finding was that poorly informed participants showed EQ-5D utility −0.23 and MCS −6.5 points compared to their well-informed counterparts. These findings emphasized the importance of tailored educational strategies to improve patients’ knowledge and, in turn, QoL. Possibly, this distinct group of patients needs accurate information provided by supportive healthcare professionals who facilitate active participation in decision-making. Healthcare systems need to first address educational deficits and learning needs across the entire PPM recipients, thus prioritizing patient-centered care. Subsequently, the evaluation should focus on identifying those who continue to perceive themselves as inadequately informed. This approach not only enables health care professionals to allocate interventions that ensure individualized support for patients who perceive informational gaps but also reduces disparities.

In terms of sex, as measured by EQ-5D-5L, male patients reported higher QoL, reflecting better perceived health status, whereas females had low QoL in Mental Component Score as measured by SF-36, reflecting greater perceived psychological distress or lower mental well-being. The EQ-5D-5L scale may underestimate domain-specific mental health disparities that the SF-36 captures more clearly. These differences may be influenced by biological, psychological, and social factors, including variations in symptom perception, coping strategies, and comorbidities. Several factors negatively affecting QoL in women include feelings of guilt related to their illness, fear of physical weakness, anxiety about dependence on others, lack of confidence in treatment efficacy, lower socioeconomic status, and higher levels of anxiety or depression [24].

Regarding age, results revealed better QoL among patients below the age of 85, as measured by the EQ-5D-5L. As a general rule, aging leads to a gradual decline in physical and mental capacities, in the ability to perform activities of daily living, and is also accompanied by retirement, loss of life purpose, death of a friend or partner, and social restrictions. Subjective symptoms experienced by patients of advanced age, such as back pain, shoulder stiffness, arthritic pain, and discomfort, are shown to reduce QoL [9]. A prior study demonstrated that the more advanced a patient’s age, the worse the QoL in terms of functional capacity [25]. A relevant study in Greece (n = 150) showed that a one-year increase in age indicates a 0.9-point decrease in physical and emotional roles, and a 0.7-point decrease in social functioning, leading to a poor QoL [2].

Patients living in Athens or other urban areas exhibited lower mental health scores compared to those residing in rural areas, as measured by the SF-36 scale. This finding may reflect the greater psychosocial stressors commonly associated with urban environments, such as higher population density, a faster pace of life, and diminished opportunities for social integration. On the other hand, the low mental health recorded by participants living in urban areas may be attributed to their greater awareness of illness due to prompt accessibility to health care services. In contrast, rural residents often benefit from stronger family bonds and community networks, which can provide emotional support and contribute to better perceived mental well-being. Notably, place of residence and geographic factors are associated with health status assessment, healthcare utilization, adequacy of available services, and health-related behaviors [26].

Employed patients reported better QoL in the EQ-5D-5L scale. Employment provides financial stability, opportunities for social engagement, a structured routine, and a sense of purpose, all of which are contributors to enhanced well-being. Unemployment is linked to financial hardship, reduced social engagement, and increased psychological stress (anxiety, loss of self-esteem), which adversely affect QoL [27].

Participants with VVI pacemakers reported lower QoL compared with those with DDD pacemakers as measured by the EQ-5D-5L. DDD pacemakers are regarded as a more physiological pacing modality that lessens atrioventricular dyssynchrony, which is associated with reduced left ventricular preload and a consequent decline in stroke volume [28]. Participants receiving DDD pacing demonstrated an improvement in utility scores by +0.12 points compared to those with VVI pacing. A possible explanation for the observed benefits of DDD pacing is its superior capacity to optimize cardiac performance by providing hemodynamic benefits over Ventricular Pacing. The increase of 0.12 in utility score represents an improved perceived health status and delivers tangible improvements that extend beyond basic cardiac rhythm management. Adoption of DDD pacing may be held as a strategy to improve patient-centered outcomes in cardiac pacing practice.

The SF-36 scale revealed that patients without rate response had lower QoL in the Physical Component compared to those with rate response. A possible explanation is that participants with no rate-responsive pacing lack the possibility for adjusting heart rate according to physiological demands and functional performance [29].

Of note, QoL domains strongly depend on the period of examination and the current circumstances. For example, globally, the COVID-19 pandemic has influenced the lifestyles of people, while the implemented restrictions have resulted in detrimental mental health consequences [30]. This study was conducted after the acute phase of the COVID-19 pandemic, but long-term effects may influence patients’ QoL, including persistent anxiety, device management, and awareness about social interactions.

In the present study, where PPM recipients with physical presence for device interrogation and clinical follow-up were included, it is important to mention the value of remote monitoring. According to Dilaveris et al. [5], remote monitoring of cardiac implantable electronic devices is gaining widespread recognition due to its associated benefits in clinical outcomes across diverse patient populations. In modern times, remote monitoring constitutes the standard of care for implanted devices and is endorsed by leading global Cardiology Organizations, including the European Society of Cardiology.

Disease-specific instrument focuses on dimensions most relevant to this patient population and may be more sensitive to clinical changes than the current generic questionnaires. However, instruments like the SF-36 and EuroQol (EQ-5D) provide a more comprehensive evaluation of general health status, capturing multiple dimensions including physical functioning, mental health, pain, and social well-being.

It is worth mentioning that the AQUAREL questionnaire, which was first developed and published in 2001 by Stofmeel et al. [31], is a disease-specific questionnaire designed for PPM recipients. The AQUAREL contains 20 items divided into three domains: chest discomfort, dyspnea on exertion, and arrhythmia. Moreover, the Global Physical Activity Questionnaire (GPAQ), developed by the World Health Organization, is a tool used to measure physical activity across work, transport, and leisure domains. It assesses frequency, duration, and intensity to classify activity levels as low, moderate, or high. Widely used in public health research, the GPAQ provides reliable data for monitoring physical activity patterns and guiding interventions to reduce sedentary behavior and related health risks [32]. Researchers or clinicians may prefer the SF-36 and EuroQol when the focus is on overall health outcomes and QoL, and, moreover, these tools are validated for a wide range of populations and can capture changes beyond physical activity (GPAQ) and symptoms after device implantation (AQUAREL).

Additional consideration should be given to other potential mediating factors, such as social support, which is a critical determinant of QoL. Close relatives like spouses or children may relieve the physical and/or emotional burden of the patient. Moreover, symptom burden, such as shoulder pain, post implantation complications, fatigue, sleep problems, and coping mechanisms were not a subject of investigation in the present study. Finally, the QoL of cardiac patients is strongly variable between hospitals, for example, urban vs. rural and public vs. private. This inter-hospital variability is attributed to several factors, such as availability and proximity of health care services, protocols for a procedure, and patients’ characteristics. Therefore, multi-center studies are essential to measure patients’ experiences and enhance the generalizability of our findings.

This study identified demographic, clinical, and device-related factors associated with QoL in pacemaker patients, addressing key objectives. However, results should be interpreted cautiously due to the cross-sectional design, single-center sample, potential biases (interviewer, selection, and measurement), and multiple testing, increasing the risk of chance findings.

## 5. Limitations of the Study

This study has several limitations that should be considered when interpreting the findings. First, the cross-sectional study limits the ability to establish causal relationships between patient characteristics, device-related factors, and QoL outcomes. Associations identified may therefore reflect correlation rather than causation. Second, the non-probability sampling (convenience sampling) of the current study is not representative of all PPM recipients in Greece, thus limiting the generalizability of the results. Third, QoL was assessed using self-reported questionnaires, which are subject to response and recall bias. Participants’ perceptions may have been influenced by current health status, mood, or social desirability, potentially affecting the accuracy of the reported QoL measures. Any non-differential measurement error in self-reported variables would likely bias associations towards the null, leading to an underestimation of true effect sizes, with a small to moderate magnitude. Moreover, the study may be affected by selection bias, as patients who agreed to participate may differ systematically from those who did not, particularly in terms of health status, educational level, or engagement with their care. This could limit the generalizability of the findings. Potential selection bias may also have resulted in an overestimation of QoL levels, as healthier or more motivated patients may have been more likely to participate. The magnitude of this bias is uncertain but potentially moderate. Additionally, some variables, such as patients’ level of information about the device, were based on subjective assessment rather than objective measures, which may introduce measurement imprecision. The subjective measurement of patients’ information level likely introduced non-differential misclassification, biasing associations toward the null and resulting in a small to moderate underestimation of true effects. The sample size was drawn from a single center; this may reduce statistical power and external validity, limiting the applicability of the results to broader pacemaker populations (single-center effect). The single center might have patients who are generally better managed than average, which would cause an overestimation of QoL and stronger positive associations. Conversely, if the center’s patients are sicker or less representative, it could cause an underestimation of QoL or weaken associations compared to the general pacemaker population. One more potential source of limitations derives from the collection of data by the method of interview, which includes the bias of the interviewer, and social desirability (interviewer and social desirability bias), with the direction of bias towards an overestimation of QoL, particularly in subjective and mental health domains. The magnitude of this bias is expected to be small to moderate, given the use of standardized and validated QoL instruments.

## 6. Conclusions

As measured by the two scales, poorer QoL was noted in participants with comorbidities, those with NYHA class II, and patients who felt poorly informed about their pacemaker. In terms of SF-36, the Physical Component was associated with the presence of rate response in the pacemaker and the Mental Component with female gender and residence in urban areas. In terms of EQ-5D-5L, patients of male gender, those below the age of 85, those with DDD pacemakers, as well as those who were employed, had better QoL.

The aforementioned results underscore the critical role of QoL among patients with implanted cardiac devices. To the best of our knowledge, QoL among PPM patients is not an extensive subject of inquiry prior to and post-operatively, or in clinical follow-up and pacemaker monitoring. Extended research may enlighten the multidisciplinary therapeutic approach for recipients. Moreover, nationwide initiatives may contribute to QoL improvement by fostering healthy behaviors among patients of all ages, thereby aligning with key-elements of healthcare outcomes.

## Figures and Tables

**Table 1:** Description of demographic characteristics (n = 120).

	n (%)
Sex	
Male	65 (54.2%)
Female	55 (45.8%)
Age	
<75 years	21 (17.5%)
75–84 years	62 (51.7%)
85+ years	37 (30.8%)
Age at PPM Implantation	
<65 years	15 (12.5%)
65–74 years	37 (30.8%)
75–84 years	50 (41.7%)
85+ years	18 (15.0%)
Occupation	
Retired	100 (83.3%)
Employed	14 (11.7%)
Homemaker/unemployed	6 (5%)
Place of Residence	
Athens or urban area	90 (75.0%)
Rural area	30 (25.0%)

**Table 2:** Description of clinical and other self-reported characteristics (n = 120).

	n (%)
Pacing mode	
VVI	21 (17.5%)
DDD	99 (82.5%)
Rate response (Yes)	93 (77.5%)
Pacemaker dependency (Yes)	43 (35.8%)
Other Comorbidities (Yes)	100 (83.3%)
Hypertension	81 (67.5%)
Atrial fibrillation	60 (50.0%)
Dyslipidemia	49 (40.8%)
Diabetes mellitus	29 (24.2%)
NYHA Class	
I	89 (74.2%)
II	31 (25.8%)
III	0 (0.0%)
Family members with chronic illness	
No	82 (68.3%)
Yes	38 (31.7%)
Do you believe your life depends on PPM?	
Very much	55 (45.8%)
Quite a lot	44 (36.7%)
A little	21 (17.5%)
Self-reported level of information about the device	
Very well informed	56 (46.7%)
Fairly well informed	50 (41.7%)
Poorly informed	14 (11.6%)
Frequency of check-ups	
Once per year	105 (87.5%)
Twice per year	15 (12.5%)

**Table 3:** Description of patients’ QoL by SF-36 (n = 120).

	Cronbach’s a	Mean (SD)	Median (IQR)
SF36			
Physical Functioning (Range: 0–100)	0.920	23.3 (16.4)	19.0 (10.0–39.0)
Role-Physical (Range: 0–100)	0.994	57.3 (21.6)	62.0 (40.0–72.0)
Bodily Pain (Range: 0–100)	0.839	64.8 (24.9)	65.0 (45.0–85.0)
Role-Emotional (Range: 0–100)	0.974	75.0 (42.4)	100.0 (66.7–100.0)
Social Functioning (Range: 0–100)	0.869	75.0 (28.9)	87.5 (50.0–100.0)
Mental Health (Range: 0–100)	0.895	73.9 (23.9)	80.0 (56.0–96.0)
General Health (Range: 0–100)	0.769	74.2 (43.6)	100.0 (12.5–100.0)
Vitality (Range: 0–100)	0.876	85.3 (20.8)	100.0 (77.5–100.0)
Component Summary Scores			
Physical Component Summary (PCS) (Range: 0–100)		38.0 (7.1)	39.0 (33.3–43.5)
Mental Component Summary (MCS) (Range: 0–100)		54.4 (14.6)	58.9 (43.9–66.6)

SD: standard deviation and IQR: interquartile range.

**Table 4:** Description of Patients’ QoL by EQ-5D-5L (n = 120).

	Cronbach’s a	Mean (SD)	Median (IQR)
EQ-5D-5L Utility (Range: −0.66 to 1)	0.848	0.8 (0.3)	0.9 (0.7–1.0)
VAS scale (Range: 0–100)		67.5 (21.5)	70.0 (50.0–80.0)

SD: standard deviation and IQR: interquartile range.

**Table 5:** Description of patients’ QoL (n = 120).

EQ-5D-5L	n (%)
Mobility	
No problems	34 (28.3%)
Slight problems	48 (40.0%)
Moderate problems	21 (17.5%)
Severe problems	14 (11.7%)
Unable to/extreme problems	3 (2.5%)
Self-Care	
No problems	99 (82.5%)
Slight problems	12 (10.0%)
Moderate problems	3 (2.5%)
Severe problems	3 (2.5%)
Unable to/extreme problems	3 (2.5%)
Usual Activities	
No problems	45 (37.5%)
Slight problems	41 (34.2%)
Moderate problems	20 (16.7%)
Severe problems	9 (7.5%)
Unable to/extreme problems	5 (4.2%)
Pain/Discomfort	
No problems	71 (59.2%)
Slight problems	23 (19.2%)
Moderate problems	23 (19.2%)
Severe problems	3 (2.5%)
Unable to/extreme problems	0 (0.0%)
Anxiety/Depression	
No problems	53 (44.2%)
Slight problems	32 (26.7%)
Moderate problems	22 (18.3%)
Severe problems	10 (8.3%)
Unable to/extreme problems	3 (2.5%)

**Table 6:** Patient characteristics associated with QoL by SF-36 scale.

Physical Component			Mental Component	
	Median (IQR)	*p*-Value	Median (IQR)	*p*-Value
Sex		0.321		0.034
Male	39.8 (35.1–42.3)		62.1 (50.1–67.4)	
Female	37.3 (31.8–43.9)		56.3 (34.0–64.8)	
Age		0.207		0.718
<75	37.7 (36.1–41.0)		56.4 (39.9–68.7)	
75–84	39.7 (35.8–43.5)		59.9 (49.9–66.5)	
85+	35.4 (29.0–43.8)		58.3 (34.9–66.0)	
Age at PPM Implantation		0.658		0.561
<65	39.2 (36.2–41.0)		63.9 (51.7–65.9)	
65–74	38.8 (35.8–41.3)		55.0 (39.9–66.5)	
75–84	40.1 (32.8–45.3)		59.9 (49.3–67.3)	
85+	36.9 (29.1–43.4)		58.9 (34.0–65.0)	
Occupation		0.905		0.890
Retired	39.0 (33.0–43.5)		59.4 (41.5–66.5)	
Employed	38.0 (36.5–40.8)		54.1 (49.5–66.8)	
Homemaker/unemployed	39.5 (28.7–41.3)		57.6 (50.3–70.2)	
Place of Residence		0.770		0.003
Athens/urban area	38.8 (34.0–43.5)		56.4 (39.9–65.0)	
Rural area	39.2 (31.8–43.4)		64.8 (59.4–69.0)	
Pacing Mode		0.111		0.064
VVI	36.8 (26.7–43.5)		55.0 (34.0–61.6)	
DDD	39.4 (34.7–43.4)		60.3 (48.2–67.3)	
Rate Response		0.046		0.570
Yes	39.0 (35.1–43.6)		58.3 (43.9–67.2)	
No	35.2 (29.3–41.2)		59.0 (38.9–63.9)	
Pacemaker Dependency		0.538		0.091
Yes	39.9 (33.5–43.7)		56.4 (41.5–64.6)	
No	38.7 (33.0–42.9)		62.0 (44.9–68.0)	
Comorbidities		0.047		0.268
Yes	39.2 (33.0–43.7)		58.3 (43.9–66.5)	
No	35.7 (34.4–39.7)		64.6 (43.5–70.2)	
ΝΥHA		0.047		0.001
Ι	39.4 (35.6–43.4)		62.2 (50.1–67.7)	
ΙΙ	34.5 (29.2–43.8)		46.8 (29.5–59.4)	
Family Members with Illness		0.955		0.964
No	38.7 (33.5–43.5)		59.4 (43.5–67.2)	
Yes	39.2 (32.4–42.7)		58.2 (49.9–65.6)	
Life Dependency on PPM		0.985		0.250
Very much	38.7 (34.0–43.7)		59.4 (49.9–68.7)	
Quite a lot	39.2 (34.2–43.2)		56.5 (43.6–66.6)	
A little	39.0 (33.0–43.4)		59.9 (39.1–64.0)	
Information about Device		0.442		0.039
Very well informed	39.5 (35.4–43.0)		60.2 (49.2–67.7)	
Fairly well informed	37.7 (32.8–43.5)		58.9 (39.1–67.2)	
Poorly informed	40.3 (30.9–43.8)		49.7 (33.9–62.2) *	
Frequency of Check-Ups		0.473		0.809
Once per year	39.0 (34.0–43.5)		59.0 (43.9–65.9)	
Twice per year	36.2 (29.2–41.2)		58.8 (37.9–69.2)	

*, statistically significantly different score than the other groups, after Bonferroni correction for multiple comparisons.

**Table 7:** Patient characteristics associated with QoL EQ-5D-5L scale.

	EQ-5D-5L (Utility)	
	Median (IQR)	*p*-Value
Sex		0.001
Male	0.9 (0.8–1.0)	
Female	0.8 (0.6–0.9)	
Age		0.019
<75	0.9 (0.8–1.0)	
75–84	0.9 (0.8–1.0)	
85+	0.8 (0.5–0.9) *	
Age at PPM Implantation		0.075
<65	0.9 (0.9–1.0)	
65–74	0.9 (0.7–0.9)	
75–84	0.9 (0.7–1.0)	
85+	0.7 (0.5–1.0)	
Occupation		0.040
Retired	0.9 (0.7–1.0)	
Employed	1.0 (0.9–1.0)	
Homemaker/unemployed	0.8 (0.6–1.0) *	
Place of Residence		0.159
Athens/urban area	0.9 (0.6–1.0)	
Rural area	0.9 (0.8–1.0)	
Pacing Mode		0.034
VVI	0.7 (0.4–0.9)	
DDD	0.9 (0.8–1.0)	
Rate response		0.188
Yes	0.9 (0.7–1.0)	
No	0.8 (0.4–1.0)	
Pacemaker Dependency		0.126
Yes	0.9 (0.7–0.9)	
No	0.9 (0.7–1.0)	
Comorbidities		0.019
Yes	0.8 (0.7–1.0)	
No	1.0 (0.9–1.0)	
ΝΥHA		0.001
Ι	0.9 (0.8–1.0)	
ΙΙ	0.6 (0.4–0.8)	
Family Members with Chronic Illness		0.760
No	0.9 (0.7–1.0)	
Yes	0.9 (0.7–1.0)	
Life Dependency on PPM		0.112
Very much	0.9 (0.8–1.0)	
Quite a lot	0.9 (0.7–1.0)	
A little/not at all	0.8 (0.6–0.9)	
Information about Device		0.005
Very well informed	0.9 (0.8–1.0)	
Fairly well informed	0.9 (0.6–1.0)	
Poorly informed	0.7 (0.2–0.9) *	
Frequency of Check-Ups		0.720
Once per year	0.9 (0.7–1.0)	
Twice per year	0.9 (0.5–1.0)	

*, statistically significantly different score than the other groups, after Bonferroni correction for multiple comparisons.

**Table 8:** Factors associated with QoL by 2 scales.

	Sf-36		EQ-5D-5L
	PCS	MCS	
Sex		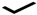	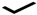
Age			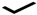
Occupation			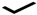
Residence		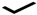	
Pacing mode			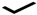
Rate Response	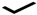		
Comorbidities	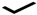		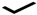
NYHA	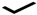	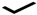	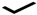
Self-reported information		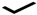	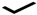

**Table 9:** Effect of patient characteristics on QoL.

	Physical Component (SF36) Adj R^2^ = 6.7% Mean VIF = 1.04		Mental Component (SF36) Adj R^2^ = 29.4% Mean VIF = 1.08		EQ-5D-5L (Utility) Adj R^2^ = 36.3% Mean VIF = 1.46	
	β Coef (95% CI)	*p*-Value	β Coef (95% CI)	*p*-Value	β Coef (95% CI)	*p*-Value
Sex(Female vs. Male)	-		−5.84 (−10.54–−1.15)	**0.015**	−0.06 (−0.14–0.02)	0.117
Age						
<75	-		-		Ref.	
75–84	-		-		0.02 (−0.10–0.14)	0.707
85+	-		-		−0.03 (−0.16–0.09)	0.599
Occupation						
Retired	-		-		Ref.	
Employed	-		-		0.06 (−0.12–0.25)	0.510
Homemaker/unemployed	-		-		−0.09 (−0.33–0.16)	0.482
Residence (Rural vs. Athens/urban)	-		10.06 (4.40–15.73)	0.001	**-**	
Pacing Mode (DDD vs. VVI)					0.12 (0.01–0.23)	0.027
Rate response (no vs. yes)	−2.40 (−5.48–0.68)	0.126	-		-	
Other Comorbidities (no vs. yes)	−2.10 (−5.95–1.75)	0.282	-		−0.04 (−0.16–0.09)	0.538
ΝΥHA (ΙΙ vs. I)	−3.33 (−6.43–−0.23)	0.036	−10.88 (−16.48–−5.27)	**0.001**	−0.20 (−0.29–−0.11)	0.001
Level of Information about Device						
Very well informed	-		Ref.		Ref.	
Fairly well informed	-		−2.07 (−7.03–2.90)	0.411	−0.08 (−0.16–0.00)	0.055
Poorly informed	-		−6.49 (−14.38–1.40)	0.106	−0.23 (−0.36–−0.11)	0.001

## Data Availability

The original contributions presented in this study are included in the article. Further inquiries can be directed to the corresponding author.
